# The analysis of tumor-infiltrating immune cell and ceRNA networks in laryngeal squamous cell carcinoma

**DOI:** 10.1097/MD.0000000000029555

**Published:** 2022-08-05

**Authors:** Dan Li, Kaifeng Dong, Jing Su, Haitao Xue, Junhai Tian, Yongfeng Wu, Jingtian Wang

**Affiliations:** a Department of Otolaryngology, The First Hospital of Hebei Medical University, Hebei ProvinceChina; b Otorhinolaryngology Surgery, The Fourth Hospital of Hebei Medical University, Hebei ProvinceChina.

**Keywords:** ceRNA networks, laryngeal squamous cell carcinoma, tumor-infiltrating immune cell

## Abstract

**Background:**

Laryngeal squamous cell carcinoma (LSCC) is one of the most common forms of head and neck cancers. However, few studies have focused on the correlation between competing endogenous RNA (ceRNAs) and immune cells in LSCC.

**Methods:**

RNAseq expression of LSCC and adjacent tissues were downloaded from The Cancer Genome Atlas to establish a ceRNA network. The key gene in ceRNA was screened by the cox regression analysis to establish a prognostic risk assessment model. The CIBERSORT algorithm was then used to screen important tumor-infiltrating cells related to LSCC. Finally, co-expression analysis was applied to explore the relationship between key genes in the ceRNA network and tumor-infiltrating cells. The external datasets were used to validate critical biomarkers.

**Results:**

We constructed a prognostic risk assessment model of key genes in the ceRNA network. As it turned out, Kaplan–Meier survival analysis showed significant differences in overall survival rates between high-risk and low-risk groups (*P* < .001). The survival rate of the high-risk group was drastically lower than that of the low-risk group, and the AUC of 1 year, 3 years, and 5 years were all above 0.7. In addition, some immune infiltrating cells were also found to be related to LSCC. In the co-expression analysis, there is a negative correlation between plasma cells and TUBB3 (*r* = −0.33, *P* = .0013). External dataset validation also supports this result.

**Conclusion:**

In this study, we found that some key genes (SLC35C1, CLDN23, HOXB7, STC2, TMEM158, TNFRSF4, TUBB3) and immune cells (plasma cells) may correspond to the prognosis of LSCC.

## 1. Introduction

Laryngeal cancer is a kind of malignant tumor of the head and neck, in which laryngeal squamous cell carcinoma (LSCC) is the most common, accounting for 96%–98%.^[1]^ The incidence of LSCC is attributed to many factors such as smoking, excessive drinking, air pollution, sex hormone levels, and viral infections.^[2]^ Recent years have witnessed the incidence of LSCC increase year by year with the acceleration of industrial processes and the aggravation of environmental pollution.^[3]^ Currently, the main treatment methods involve surgery, chemotherapy, and radiotherapy. However, the complications from these treatments, as well as relapses and metastasis affecting the prognosis, can seriously interfere with a patient’s normal life.^[4]^ Therefore, early prevention, diagnosis, personalized treatment, and the search for precise targeted therapeutic drugs are of great significance to increase the survival rate of patients. The treatment of LSCC requires the selection of appropriate treatment options according to the patient’s clinical stages, metastasis ranges, tumor sizes, and ages. Surgical treatments are often applied for early LSCC, including total laryngectomy, partial laryngectomy, oral laser microsurgery, etc., quickly removing the lesions and effectively controlling the disease.^[5]^ Unfortunately, the early symptoms of LSCC are not significant, and most patients are diagnosed in stage III or IV.^[6]^ At this stage, the treatment effect of patients is not satisfactory to a certain extent.

Previous research presents a competing endogenous RNAs (ceRNA) hypothesis.^[7]^ lncRNAs competitively bind to miRNAs, to regulate the expression level of mRNAs and involve in the regulation of biological behaviors of tumor cells.^[8–10]^ The ceRNA network plays a vital role in the development of various malignant tumors.^[11–13]^ A large number of studies have shown that miRNA can guide RNA-induced silencing complex (RISC) to bind to target mRNA, leading to RNA degradation or translational inhibition.^[14]^

In recent years, tumor immune infiltrating cells have attracted widespread attention, especially in immunotherapy.^[15]^ LSCC is rich in tumor immune infiltrating cells, and most patients respond positively to immunotherapy.^[16]^ Some studies have shown that the different compositions and locations of tumor immune cells are closely related to the prognosis of LSCC.^[17,18]^ However, in past studies, researchers used traditional methods, such as immunohistochemistry, to explore the composition of immune cells in malignant tumor tissues. The number of cells that these methods can detect is very limited.^[19]^ With the development of various omics databases, some new methods for detecting immune cells based on machine learning have been born. For example, CIBERSORT, can estimate the abundance of 22 immune cell types from gene expression profiles.^[20]^ Many studies use it to analyze the proportion of immune cells in cancer.^[21,22]^

Previous studies have separately reported the role of the ceRNA network and tumor immune cells in LSCC.^[23,24]^ So far, there are only a few papers to comprehensively study the functions of ceRNAs and tumor immune cells in LSCC. Therefore, in this study, we hope to perform a co-expression analysis between ceRNAs and immune cells to identify potential immune-related biomarkers.

**Figure 1.**
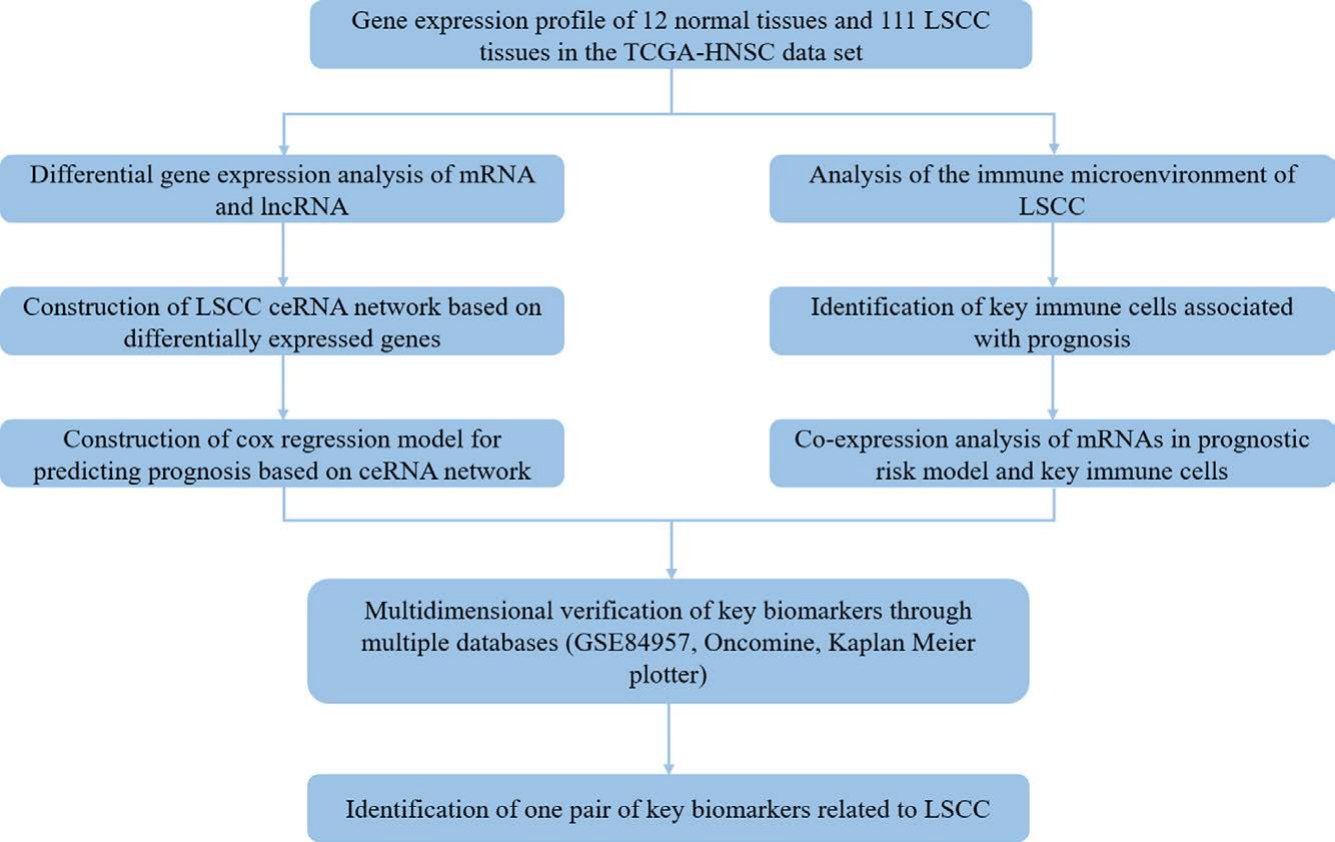
The experimental flowchart of this study.

In this study, a ceRNA network for LSCC was established, which is determined by gene expression in the Cancer Genome Atlas (TCGA) database. The CIBERSORT was used to evaluate the proportion of immune cells in LSCC samples and quantify the cellular composition of the immune response. Afterward, genes in the ceRNA prognostic model and the key immune cells that affect the prognosis were screened. Co-expression analysis of key genes and immune cells was carried out to explore the potential mechanisms affecting the prognosis of LSCC. As it turned out, these findings may provide new ideas for the prediction and treatment of LSCC. We show the experimental flowchart in Figure 1.

## 2. Materials and Methods

### 2.1. Data selection and analysis of differential gene expression

The RNAseq data of 123 patients with LSCC and normal samples were obtained from The Cancer Genome Atlas (TCGA) (Version October 21, 2020) (including 111 cases of cancer tissue and 12 cases of normal tissue adjacent to cancer) (https://cancergenome.nih.gov/).^[25]^ The clinical data was downloaded by the Xena browser (https://xenabrowser.net/). Based on Ensembl annotation (http://www.ensembl.org), RNAseq data were divided into lncRNA and mRNA expression matrices.^[26]^ Besides, the demographic information for each patient (age, gender, survival status, number of days of death, tumor stage, etc.) was downloaded. With R software and edgeR package, differentially expressed mRNAs (DEmRNAs) and differentially expressed lncRNAs (DElncRNAs) were obtained, and volcano maps and heat maps were drawn. The selection criteria for DEmRNAs and DElncRNAs is |logFC|> 2.0, *P* < .01.^[27]^

### 2.2. Construction of the ceRNA network

The mircode database (http://www.mircode.org) is used to identify the interactions between lncRNA and miRNA.^[28]^ Then, miRNA target genes could be searched from miRDB databases.^[29–31]^ After determining lncRNA-miRNA pairs and miRNA-mRNA pairs, we examined DEmRNAs and DElncRNA obtained from the edgeR package, respectively. Only lncRNA-miRNA and miRNA-mRNA pairs formed by differentially expressed RNA were screened. Eventually, we used Cytoscape v3.7 to construct the lncRNA-miRNA-mRNA network. The mRNA in the ceRNA network is the part that performs biological functions. Hence, the Gene Ontology (GO) and Kyoto Encyclopedia of Genes and Genomes (KEGG) enrichment analysis of these genes was performed to understand the biological functions of the network. The metascape database was used for mRNA enrichment analysis (https://metascape.org/gp/index.html#/main/step1).

### 2.3. Construction of prognostic risk model related to the ceRNA network

The expression data of each DEmRNA and DElncRNA in the ceRNA network was extracted, and we used the survival data of each sample for single-variable COX regression analysis. The survival cox ph feature in R software was used and log-rank *P* < .05 was chosen as the threshold for screening key genes that affect prognosis. Furthermore, the glmnet package in the R software is used to perform a set of cox regression analyses. After that, the prognostic risk assessment model of the ceRNA network is constructed with multivariable COX regression analysis. Based on the medium-risk score, we calculated the risk score for each sample separately and divided the patients into high-risk and low-risk groups, respectively. The Kaplan–Meier method was applied to analyze the difference in overall survival (OS) between 2 groups. Use the timeROC package of R software to draw ROC curves of 1, 3, and 5 years.

### 2.4. CIBERSORT estimation

The abundance of 22 different types of immune cells was estimated using CIBERSORT in R software (Version 4.0.2).^[20]^ Wilcoxon rankings test identifies differences in immune infiltration between normal samples and patient samples. The result of *P* < .05 indicates that the difference is statistically significant. In addition, corrplot and vioplot packages in R were also applied to visualize the results. The effects of immune cells on prognosis were analyzed by the Kaplan–Meier method.

### 2.5. Co-expression analysis of mRNAs in prognostic risk model and key immune cells

The corrplot package in R can be used for correlation analysis of 7 mRNAs and key immune cells. Under the Pearson correlation analysis, a co-expression heatmap was graphed to show the correlation between mRNAs and immune cells. The ggplot package in R was used to plot the correlation curves for mRNAs and immune cells that are highly correlated.

### 2.6. Multidimensional validation

The Gene Expression Omnibus (GEO) database is that of gene expression created and maintained by the National Center for Biotechnology Information. Moreover, it contains high-throughput gene expression data submitted by research institutions around the world.^[32]^ The GSE84957 data set was used to confirm the expression of key genes in normal tissues and cancer tissues. All patients were provided written informed consent before their participation. The study was undertaken per the Institutional Ethics Committee of Beijing Tongren Hospital Affiliated with Capital Medical University and the ethical standards of the World Medical Association Declaration of Helsinki.^[33]^ Kaplan Meier-plotter (http://kmplot.com/analysis/index.php?p=service) is a website for online survival analysis. Currently, the website has researched 54,675 genes and 18,674 cancer samples, involving breast cancer, lung cancer, gastric cancer, etc. Based on the Kaplan Meier-plotter website, it is used for survival analysis of critical mRNA. It can verify whether there is a significant difference in the survival time of patients at different expression levels.^[34]^ In addition, oncomine was used to analyze the differential expression of key mRNAs in histological types of tumors and normal tissues (https://www.oncomine.org/resource/login.html#).^[35]^

## 3. Results

### 3.1. Screening of differentially expressed genes and construction of the ceRNA network

**Figure 2.**
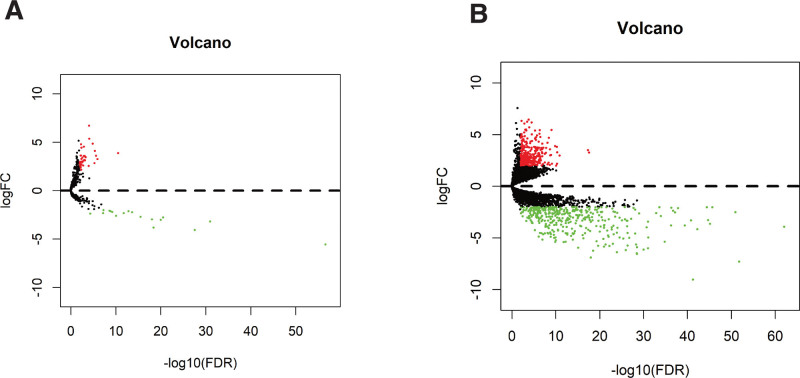
(A) Volcano map based on lncRNA expression values of 123 samples in TCGA database. Green represents low expression and red represents high expression. (B) Volcano map based on mRNA expression values of 123 samples in TCGA database. Green represents low expression and red represents high expression.

**Figure 3.**
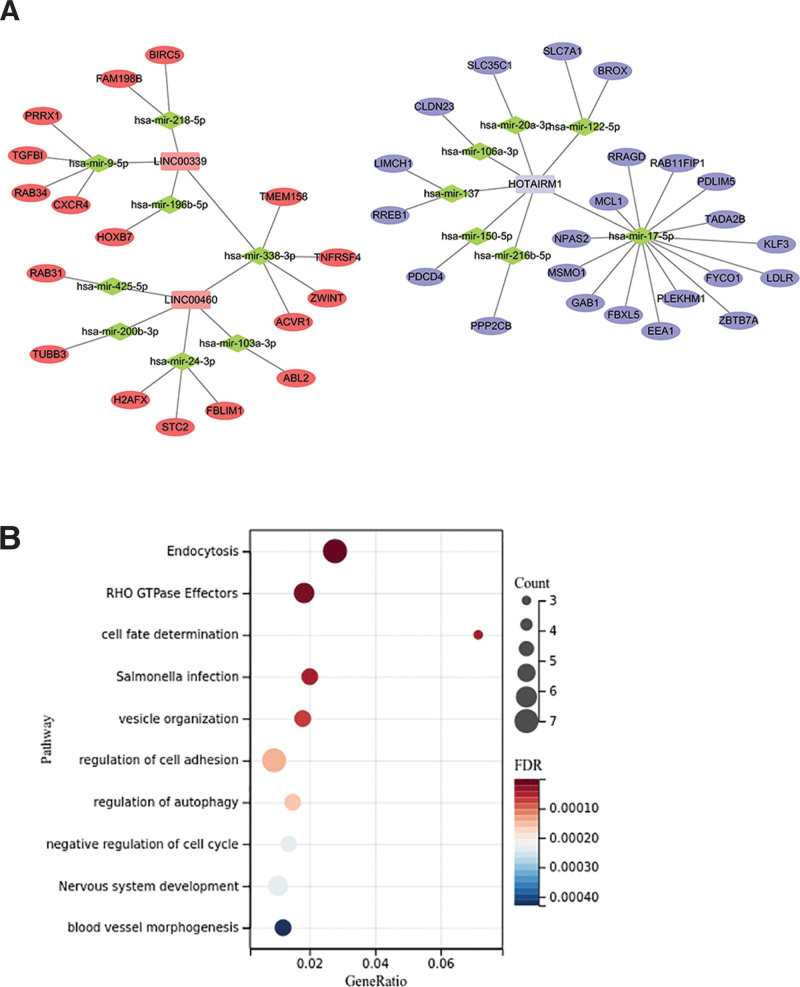
(A) Construction of the ceRNA network related to LSCC. Square nodes represent lncRNAs, where red is upregulated lncRNA and blue is downregulated lncRNA. Diamond-shaped nodes represent miRNAs. Oval nodes represent mRNAs, where red is upregulated mRNA and blue is downregulated mRNA. (B) Enrichment analysis of mRNAs in the ceRNA network.

There are 111 LSCC and 12 adjacent samples in the TCGA head and neck squamous cell carcinoma (HNSCC) dataset (Table 1). As shown in Figure 2, there are 662 DEmRNAs (289 mRNA upregulated, 373 mRNA downregulated) in LSCC samples; 57 DElncRNAs (39 upregulated, 18 downregulated) compared with adjacent tissues. Among these differentially expressed RNAs, we constructed a ceRNA network containing DElncRNAs and DEmRNAs, revealing the complex competition and connections between endogenous RNAs. We took advantage of Cytoscape 3.7.0 software to construct a ceRNA network with 58 nodes and 56 edges, including 3 lncRNAs, 15 miRNAs, and 40 mRNAs. The relationship between the different forms of RNA is illustrated in Figure 3A.

**Table 1:** Clinical information statistics of TCGA dataset.

Characteristic		TCGA dataset
Survival status	Alive	67
	Dead	50
Age	≤60	49
	>60	68
Sex	Female	20
	Male	97
Grade	G1	8
	G2	72
	G3	32
	G4	1
T	T1	7
	T2	14
	T3	26
	T4	55
N	N0	41
	N1	12
	N2	41
	N3	2
M	M0	41
	M1	9
Stage	I	2
	II	10
	III	14
	IV	74

Based on the metascape database, we carried out GO and KEGG enrichment analyses on mRNAs that performed biological functions in ceRNA (Figure 3B). The results indicate that these biological processes are mainly concentrated in endocytosis, RHO GTPase effectors, cell fate determination, salmonella infection, vesicle organization, regulation of cell adhesion, regulation of autophagy, negative regulation of cell cycle, nervous system development, blood vessel morphogenesis.

### 3.2. Analysis of prognostic risk model related to ceRNA network

**Figure 4.**
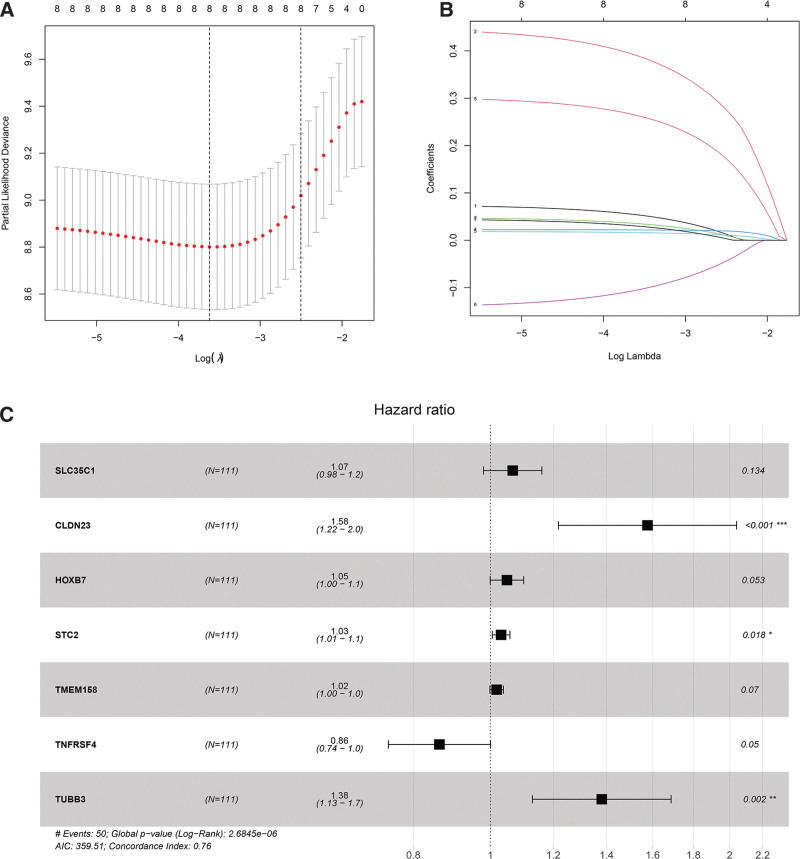
(A) LASSO model after 10-fold cross-validation. The 2 vertical dashed lines represent lambda.min and lambda.lse respectively. (B) Coefficient distribution of LASSO model. (C) Forest plot of the prognostic risk model of key genes in the ceRNA network. * means that the difference is statistically significant.

First, we perform a univariate COX proportional hazard regression model on differentially expressed genes and survival data in the ceRNA networks. The results showed that 9 differentially expressed genes had a significant effect on the prognosis, all of which were mRNAs. We made use of the glmnet package in R to perform lasso cox regression analysis. In the first step, the change trajectory of each independent variable is displayed in Figure 4A. As the lambda increases, independent variable coefficients tend to gradually increase. We exploited a 10-fold crossover method to test the model, and then analyze the confidence interval for each lambda (Figure 4B). When lambda=0.013748, the model is optimal. The model at this point encompasses 8 mRNAs. Furthermore, we performed a multivariate cox regression analysis on the 8 mRNAs obtained in the previous step and retained the 7 mRNAs with the minimum AIC value (AIC = 359.51) as the final model (Figure 4C). The final 7-mRNA signature formula is as follows.

RiskScore=0.065*expSLC35C1 + 0.455*expCLDN23 + 0.048*expHOXB7 + 0.031*expSTC2 + 0.018*expTMEM158-0.148*expTNFRSF4 + 0.323*exp TUBB3

**Figure 5.**
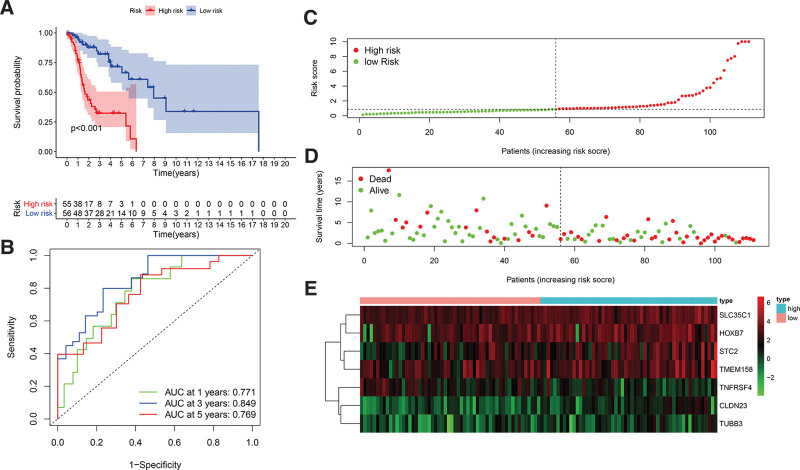
(A) The overall survival curve of high-risk and low-risk patients based on Kaplan-Meier analysis. (B) 1-year, 3-year, and 5-year ROC curves based on prognostic model. (C) Distribution of risk scores for patients with LSCC. (D) Risk score and survival status of patients with LSCC. (E) Heatmaps of key mRNA expression values in samples of the high-risk and low-risk groups.

To evaluate the prognostic effect of the model, the samples were divided into high-risk groups and low-risk groups. As is shown in Figures 5C–E, the distribution of risk scores based on the 7-mRNA signature model and their corresponding expression profiles in the TCGA-HNSCC dataset. Moreover, based on the analysis of the prediction accuracy rates of the model for 1, 3, and 5 years according to RiskScore, it can be seen that the model has a large area under the curve (AUC), and the AUC for 1, 3, and 5 years are all above 0.7(Figures 5B). Kaplan–Meier survival analysis suggests the significant differences in the overall survival rate of the high-risk groups and the low-risk groups (*P* < .001). The survival rate of patients in the high-risk group was significantly lower than that of the low-risk group (Figures 5A). These results indicate that RiskScore can effectively screen high-risk patients with poor clinical prognosis.

### 3.3. The composition of tumor-infiltrating immune cells of LSCC

**Figure 6.**
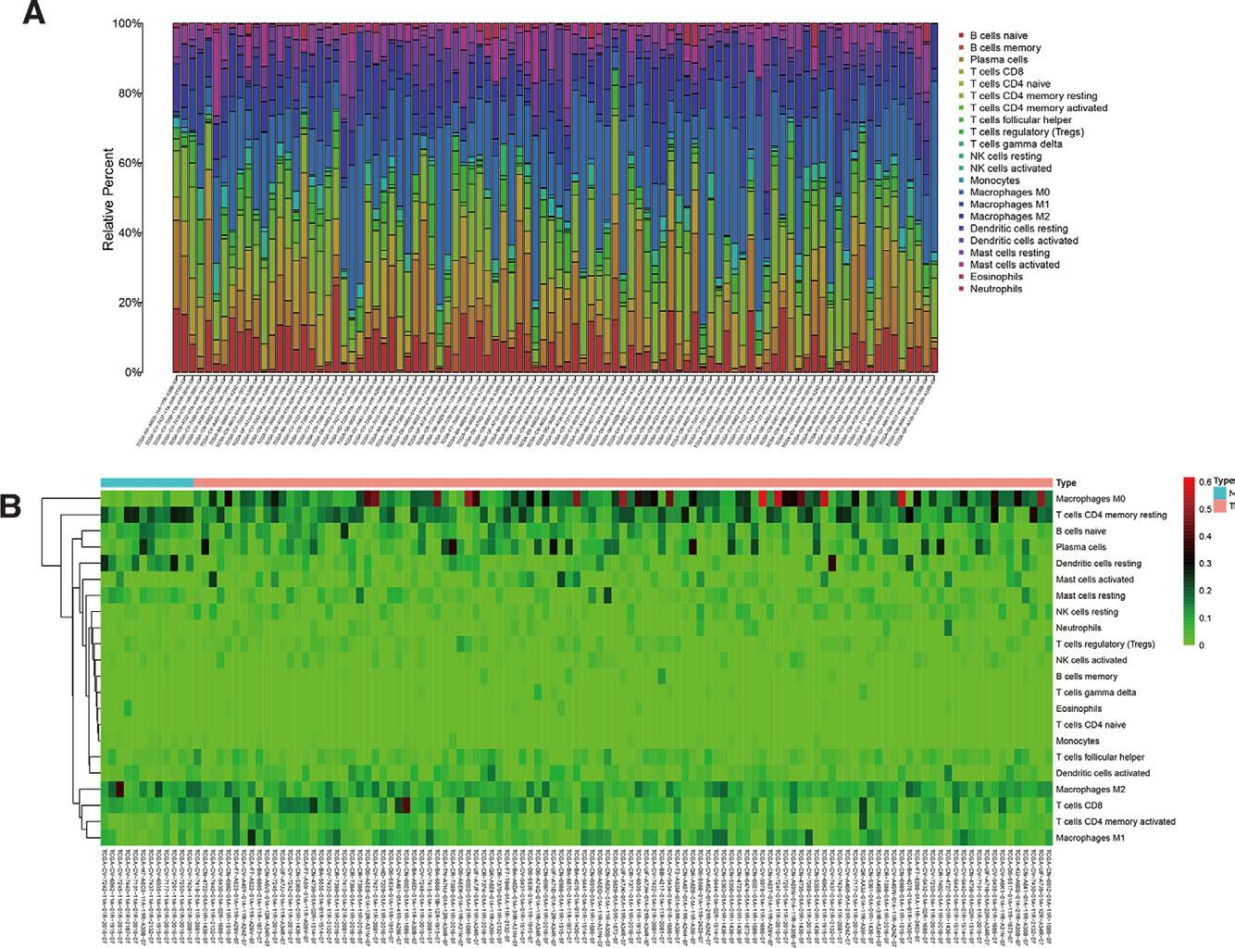
(A) Histogram of immune cell infiltration in TCGA samples. The abscissa represents the sample name, and the ordinate represents the percentage of immune cells. Different colored bars represent different immune cells. (B) Heatmap of immune cell infiltration in each sample. The abscissa is the sample name, and the ordinate is 22 immune cells. The shade of color represents the relative expression of immune cells.

**Figure 7.**
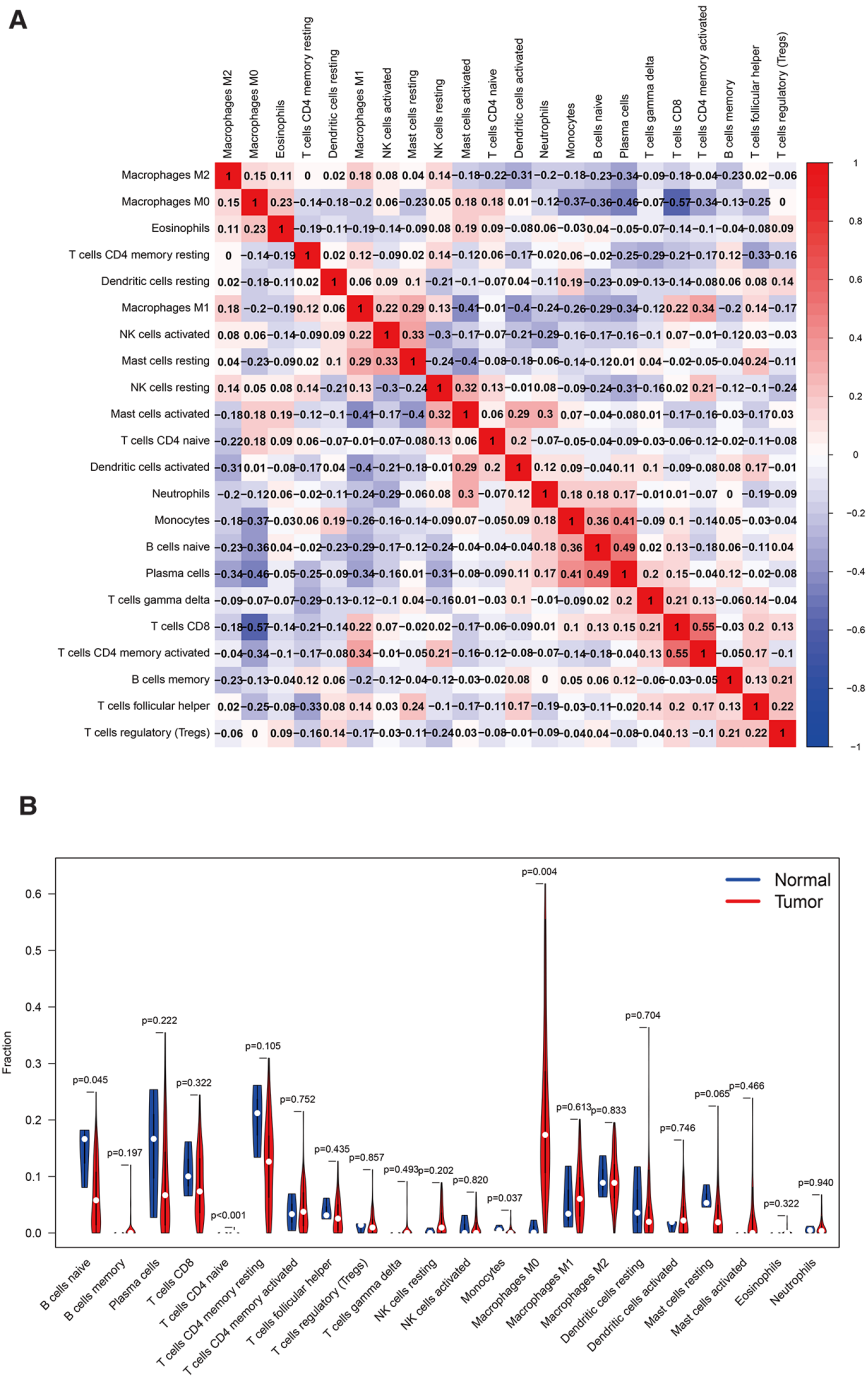
(A) Correlation heatmap of 22 immune cells. The color of the square represents the correlation between the 2 immune cells. (B) The expression difference of 22 immune cells in normal samples and patient samples. The blue violin column represents the normal sample, and the red violin column represents the patient sample. The p-value of the 2 sets of samples after the rank-sum test is located above the violin column.

**Figure 8.**
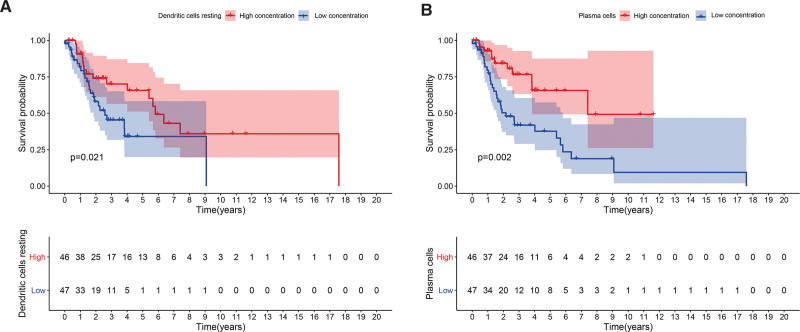
(A)The influence of Dendritic cells resting on the prognosis of patients with LSCC. (B)The influence of plasma cells on the prognosis of patients with LSCC.

The CIBERSORT algorithm was used to estimate the abundance of 22 immune cells. The distribution of tumor-infiltrating immune cells in normal and patient samples is shown in Figure 6A. It shows immune cell types and relative percent in LSCC tissues. Figure 6B is the heatmap of tumor-infiltrating cells in LSCC tissues and control group tissues. The correlation analysis of immune cells showed that Monocytes, B cells naive and plasma cells were positively correlated. T cells CD8 and T cells CD4 memory activated are positively correlated. Monocytes, B cells naïve, plasma cells, and T cells CD8 are all negatively correlated with Macrophages M0. Furthermore, Dendritic cells activated and Mast cells activated are negatively correlated with Macrophages M1(Fig. 7A). In the violin chart (Fig. 7B), the Wilcoxon rank-sum test showed that B cells, Monocytes, and Macrophages M0 in normal tissues and tumor tissues are different. B cells and monocytes are present at lower concentrations in tumors while M0 macrophages are more abundant. The survival analysis showed that Dendritic cells resting (*P* = .021) and plasma cells (*P* = .002) may be correlated with the prognosis of LSCC patients (Fig. 8).

### 3.4. Co-expression analysis of mRNAs in prognostic risk model and key immune cells

**Figure 9.**
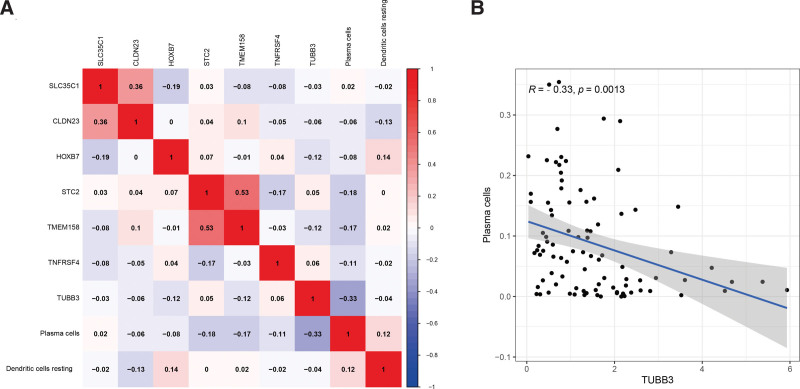
(A) Correlation analysis between key members of ceRNA network and key members of immune cells. (B) Scatter plot of the correlation between TUBB3 and plasma cells.

To explore the correlation between key RNAs and prognosis-related immune cells, we performed a Pearson correlation analysis (Fig. 9). The results showed significant correlations between SLC35C1 and CLDN23(*R* = 0.36, *P* < .001), STC2 and TMEM158(*R* = 0.53, *P* < .001), and TUBB3 and plasma cells (*R* = −0.33, *P* = .0013). Further co-expression analysis also revealed a significant positive correlation between SLC35C1 and CLDN23, STC2 and TMEM158, and a significant negative correlation between TUBB3 and plasma cells.

### 3.5. Multidimensional validation

**Figure 10.**
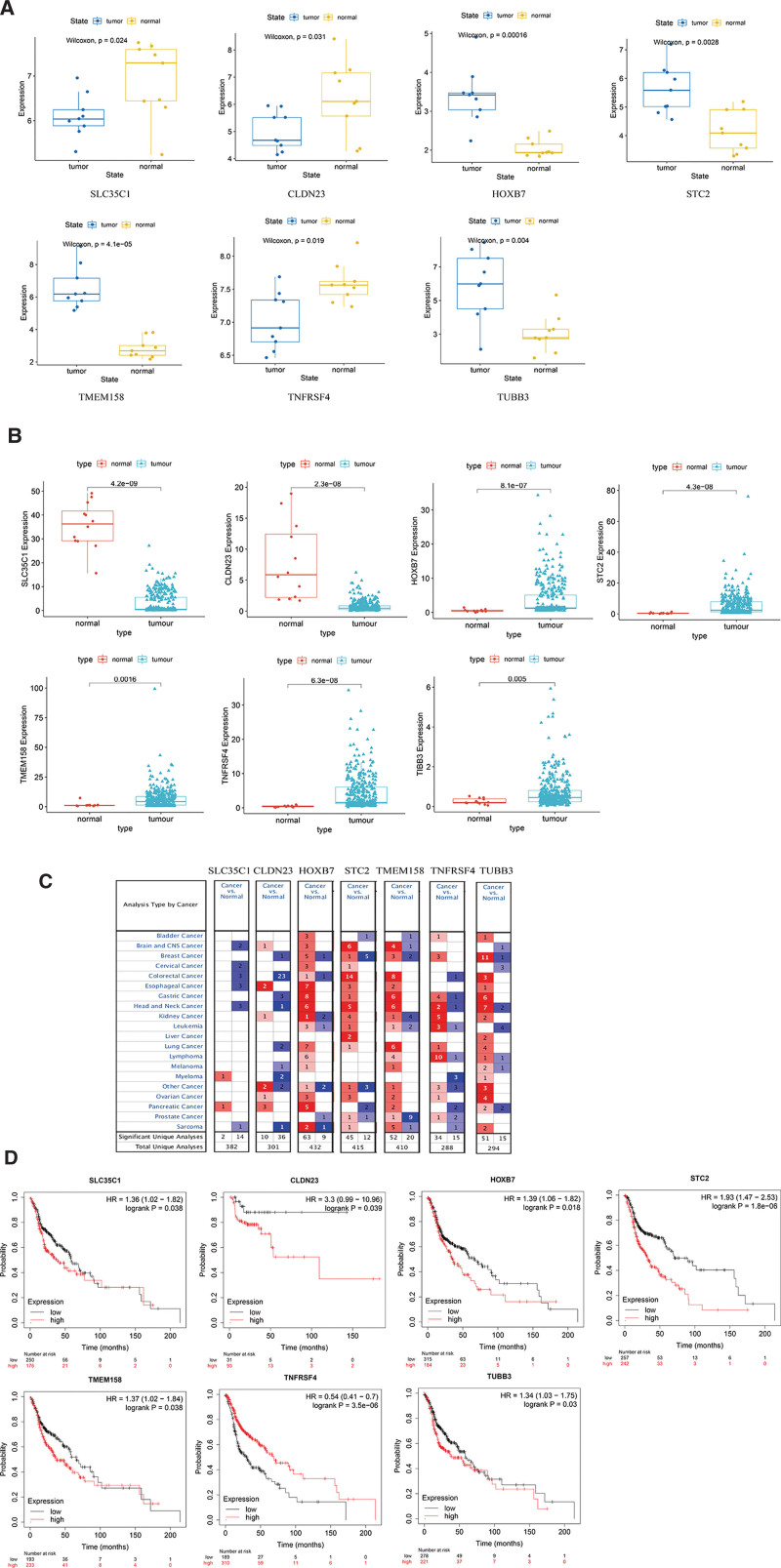
(A) Based on the GSE84957 data set, the expression of key mRNA in LSCC and normal samples was verified. The box plot shows mRNA expression in laryngeal carcinoma (blue) and corresponding normal tissues (yellow). (B) Based on the TCGA database, the expression of key mRNA in LSCC and normal samples was verified. The box plot shows mRNA expression in laryngeal carcinoma (blue) and corresponding normal tissues (red). The numbers in the table represent the number of studies included in the oncomine database. (C) Based on the oncomine database, identify the expression of key mRNA in different tumors. Red and blue represent upregulation or downregulation, respectively. (D) Based on the Kaplan–Meier database, the key mRNA in the model was validated for the prognosis of LSCC.

The GSE84957 microarray in the GEO database contains 9 primary LSCC tissues and 9 corresponding adjacent non-tumor tissues. For that matter, it was used to verify the expression levels of 7 key genes. Admittedly, compared with normal tissues, SLC35C1, CLDN23 and TNFRSF4 were significantly downregulated in LSCC tissues (*P* < .05), HOXB7, STC2, TMEM158, and TUBB3 were significantly upregulated in LSCC tissues (*P* < .05, Figure 10A). In the TCGA database, compared with normal tissues, SLC35C1 and CLDN23 were significantly downregulated in LSCC tissues (P < .05), and HOXB7, STC2, TMEM158, TNFRSF4, and TUBB3 were significantly upregulated in LSCC tissues (*P* < .05, Figure 10B). The expression level of TNFRSF4 in GEO and TCGA databases is inconsistent. According to previous studies, the expression level of TNFRSF4 is controversial. Both high and low expressions of TNFRSF4 have been reported in tumor tissues.^[36,37]^

We used the oncomine database to analyze the expression levels of key genes for different types of cancer (oncomine parameter selection *P* value <.01, logfc>1.5, generank selection top 10%). As shown in Figure 10C, red boxes indicate high expression and blue boxes indicate low expression. The numbers in the table represent the number of studies included in the oncomine database. The Kaplan–Meier method was further used to analyze critical mRNAs to assess their impact on overall survival (Figure 10D). Among them, the high expressions of SLC35C1, CLDN23, HOXB7, STC2, TMEM158, and TUBB3 are all related to patients with poor prognostic (*P* < .05), and the high expression of TNFRSF4 is related to the better one (*P* < .05). In the calculation results of TCGA and GEO, HOXB7, STC2, TMEM158, and TUBB3 genes are all highly expressed in patients. This is consistent with their high expression and poor prognosis. TNFRSF4 is low expression in patients, which is consistent with high expression and better prognosis in patients.

## 4. Discussion

After the differentially expressed ceRNA network between LSCC and adjacent tissues and the key immune cells in the tumor microenvironment has been identified, we constructed a prognostic prediction model based on the selected ceRNA network and immune cells. In this regard, SLC35C1, CLDN23, HOXB7, STC2, TMEM158, TNFRSF4, and TUBB3 in the model can effectively predict the prognosis. SLC35C1 and CLDN23 are downregulated in most cancers.^[38–41]^ HOXB7, STC2, TMEM158, and TUBB3 are upregulated in most cancers (Figure 10C). For example, HOXB7 is highly expressed in breast cancer, ovarian cancer, and melanoma samples.^[42–45]^ STC2 is highly expressed in rectal cancer and colon cancer.^[46,47]^ TMEM158 is upregulated in laryngeal cancer, glioblastoma, and colorectal cancer.^[48–50]^ TUBB3 is upregulated in gastric cancer, gallbladder cancer, and ovarian cancer.^[51–53]^ STC2 is the encoding gene for stanniocalcin-2. One study investigated the expression of STC2 in 70 esophageal cancer cell lines. The expression of STC2 in cancer tissues was higher than in the corresponding normal tissues (*P* < .001). Additionally, STC2 expression was significantly associated with lymph node metastasis, lymphatic invasion, and long-range metastasis (*P* = .005, .007, and .038, respectively). Obviously, the 5-year survival rate of patients with high STC2 expression was lower than those with low STC2 expression rates (*P* = .016). In vitro experiments showed that the proliferation rate of STC2 transfected cells was significantly higher than that of control cells (*P* < .001). STC2 transfected cells were also more aggressive than the control cells (*P* < .001).^[35]^ TMEM158 is a gene that encodes transmembrane protein 158. Its upregulation promotes the progression of several cancers. TMEM158 was significantly upregulated in pancreatic cancer samples. Studies of the mechanism have demonstrated that the activation of TGFβ1 and PI3K/AKT signals may be the aggressive cause of TMEM158 triggering pancreatic cancer. TGFβ1 has the effect of promoting carcinogenesis. A study demonstrated that TMEM158 was an upstream regulator of TGFβ1 by western blot, qRT-PCR, and ELISA experiments. Blockade of TGFβ1 significantly reversed TMEM158 overexpression-induced pancreatic cancer cell metastasis and epithelial-mesenchymal transition. PI3K/AKT signaling has been widely implicated in cancer cell proliferation, metastasis, and apoptosis. TMEM158 was able to stimulate increased PI3K/AKT signaling in pancreatic cancer cells. Inhibiting the expression of TMEM158 can effectively reverse the pancreatic cancer cell proliferation, migration, and invasion induced by PI3K/AKT signaling.^[54]^ SLC35C1 is a GDP fucose transporter negatively regulating the WNT signal pathway. In HEK293 cells, the silence of SLC35C1 can activate the WNT pathway, while the hyperexpression of SLC35C1 suppresses this pathway. WNT plays an important role in the maintenance of homeostasis, and abnormal activation of the WNT pathway is associated with a variety of cancers. SLC35C1 is a negative regulator for the WNT signaling pathway. Its abnormal expression may lead to over-activation of WNT signaling in cancer cells.^[55]^ CLDN23 is the encoded gene of claudin-23. CLDN23 showed low expression in tumor patient samples in GEO and TCGA databases. The expression of CLDN23 is also downregulated in other types of tumors.^[41]^ For example, intestinal tumors can reduce the expression of CLDN23. CLDN23 is reduced in tumor tissue compared to nearby normal mucosa.^[56]^ However, survival analysis showed that low expression of CLDN23 was associated with longer OS. In previous studies, the relationship between CLDN23 and OS was controversial. Studies have reported that lower CLDN23 mRNA levels are associated with poorer OS.^[40,57]^ There is also a Cox multivariate survival analysis showing that when CLDN23 is low expressed, the OS of gastric cancer patients is longer.^[58]^ A recent study showed that low expression of CLDN23 was associated with longer OS in colorectal cancer patients of CMS4 and C4 subtypes. In contrast, in the CMS2 and C1 subtypes, low CLDN23 expression was associated with shorter OS. It was shown that CLDN23 plays a dual role as a tumor suppressor/promoter in colorectal cancer.^[59]^ Therefore, the effect of CLDN23 on prognosis is controversial, possibly because CLDN23 in different subtypes has different effects on prognosis, and our study did not divide the samples into subtypes. HOXB7 is the coding gene of homeobox protein Hox-B7. Previous studies have proved that HOXB7 activation may be a functional bridge between the homeobox gene and tumor progression. Besides, HOXB7 can also induce other genes to be directly or indirectly related to angiogenesis and tumor invasion. Vascular endothelial growth factors, interleukin-8, and angiopoietin-2 can all be upregulated by HOXB7 transduction.^[60–62]^ In a study of patients with pancreatic cancer, HOXB7 mRNA and protein levels increased significantly in pancreatic ductal adenocarcinoma cell lines and patient tumor samples compared to normal samples. Tissue microarray evaluation of 145 pancreatic ductal adenocarcinoma samples revealed that high expression of HOXB7 protein was associated with lymph node metastasis (*P* = .034), which resulted in poor prognostication in patients. Knocking out or overexpression of HOXB7 in pancreatic ductal adenocarcinoma cell lines leads to decreased or increased invasiveness, respectively. HOXB7, together with its downstream targets may become new clinical biomarkers or therapeutic targets.^[63]^ TUBB3 is the gene encoding the tubulin beta-3 chain. Overexpression of TUBB3 has been found to be related to the poor prognosis of some solid tumors including HNSCC. A study performed immunohistochemical staining on 667 cases of oral cancer, hypopharyngeal cancer, and LSCC tissues to detect the expression of TUBB3. It was demonstrated that more than 90% of tumors showed clear cytoplasmic TUBB3 expression. 69 cases (15.5%) were weakly positive, 149 cases (33.5%) were moderately positive, and 188 cases (42.2%) were strongly positive.^[64]^ Other studies have shown that some miRNAs, such as miR-200b-3p, can regulate the resistance of colorectal cancer cells to oxaliplatin by targeting TUBB3. It may be a potential drug target for colon cancer.^[65]^ However, there are few studies on these genes in LSCC.

Dendritic cells are the most powerful professional antigen-presenting cells. Mature dendritic cells can effectively activate the initial T cells, at the center of initiating, regulating, and maintaining the immune response. A dendritic cell is closely related to the occurrence and development of tumors. A large number of dendritic cells in most solid tumors results in a good prognosis. The cellular immune response dominated by CD8+ T cells is the basis of dendritic cells as an immunotherapy method.^[66]^ Dendritic cells can induce high expression of major histocompatibility complex I and major histocompatibility complex II molecules. Major histocompatibility complex molecules bind to tumor antigens to form peptide- major histocompatibility complex molecular complexes. It is then presented to T cells to initiate the major histocompatibility complex I restricted cytotoxic T lymphocyte response and the major histocompatibility complex II-restricted CD4+ Th1 response.^[67]^ At the same time, dendritic cells also provide the second signal required for T cell activation through costimulatory molecules (CD80/B7-1, CD86/B7-2, CD40, etc.) to initiate an immune response.^[68]^ The combination of dendritic cells and T cells can secrete a large amount of IL-12 and IL-18 to activate T cell proliferation, which is conducive to tumor clearance. Dendritic cells can secrete chemotactic cytokines and upregulate the expression of IL-12, CD80, and CD86.^[69]^ In addition, Dendritic cells also present antigen peptides directly to CD8+ T cells. CD4+ and CD8+ T cells can further enhance the anti-tumor immune response by secreting cytokines.

Plasma cells, also called effector B cells, are cells in the immune system that release large amounts of antibodies. It was indicated in a retrospective study that in 69 studies of 19 cancers, 50.0% of patients reported a positive effect of plasma cells on prognosis, while the rest had a neutral (40.7%) or negative (9.3%) effect. When plasma cells are present, the prognostic effect of T cells is generally stronger.^[70]^ In addition, 21 studies inferred the proportion of plasma cells from gene expression data, most of which presented positive predictive effects. There is plenty of evidence to support the positive role of plasma cells in anti-tumor immunity.^[71]^ Our results indicate that high concentrations of dendritic cells and plasma cells are associated with a better prognosis. This is consistent with previous reports in the literature.

In the correlation analysis, a significant positive correlation was found between SLC35C1 and CLDN23, STC2 and TMEM158, and a significant negative correlation between TUBB3 and plasma cells. From our research, a high level of plasma cells can make patients have a better prognosis. In tumor tissues, TUBB3 was significantly higher than that in adjacent tissues, while patients with high levels of TUBB3 were at higher risk. The results of the final analysis are consistent with the results of these studies. Plasma cell and TUBB3 (*R* = −0.33, *P* = .0013) showed a significant negative correlation. Therefore, we reasoned that plasma cells and TUBB3 may play critical roles in the progression of LSCC. However, after a systematic literature review, there are no relevant biological experiments to support our computational results. We will continue to explore the underlying mechanisms by which plasma cells and TUBB3 affect LSCC in future work.

Inevitably, some related limitations and shortcomings must be acknowledged. First of all, the amount of data collected from public databases is limited, in that the clinical samples analyzed in our research are relatively incomplete. At present, despite the rapid development of omics technology, a large number of research projects on detecting activity indicators are already feasible.^[72]^ Large-scale experimental data is still difficult to obtain with very expensive testing costs as well.^[73]^ Last but not least, the biggest problem in this study is the lack of validation of key genetic mechanisms. However, in order to reduce this bias, we also used multiple databases to reveal the gene expression of key biomarkers in tumors and adjacent tissues.

## 5. Conclusion

As for differentially expressed mRNAs and lncRNAs, a ceRNA prognostic risk model was constructed to predict survival and prognosticity in patients with LSCC. The higher AUC value proves the accuracy of our model. We have identified HOXB7, STC2, TMEM158, TUBB3, and other key genes related to the prognosis from the ceRNA network. Immune microenvironment analysis found that high levels of Dendritic cells and plasma cells will give patients a better prognosis. The co-expression analysis also illustrated that plasma cells and TUBB3 are related, which suggested that they may jointly affect the prognosis of LSCC patients.

## Acknowledgments

The authors gratefully acknowledge the support of this work by the Scientific Research Fund Project of the Hebei Provincial Health Commission (No. 20201146).
